# A systematic approach for peptide characterization of B-cell receptor in chronic lymphocytic leukemia cells

**DOI:** 10.18632/oncotarget.17076

**Published:** 2017-04-13

**Authors:** Paula Díez, Nieves Ibarrola, Rosa M. Dégano, Quentin Lécrevisse, Arancha Rodriguez-Caballero, Ignacio Criado, Wendy G. Nieto, Rafael Góngora, Marcos González, Julia Almeida, Alberto Orfao, Manuel Fuentes

**Affiliations:** ^1^ Department of Medicine and General Cytometry Service-Nucleus, Cancer Research Centre (IBMCC/CSIC/USAL/IBSAL), 37007 Salamanca, Spain; ^2^ Proteomics Unit, Cancer Research Centre (IBMCC/CSIC/USAL/IBSAL), 37007 Salamanca, Spain; ^3^ Hematology Service, Institute of Biomedical Research of Salamanca, University Hospital of Salamanca, Cancer Research and Institute of Molecular Biology and Cellular Oncology, 37007 Salamanca, Spain

**Keywords:** B-cell receptor, chronic lymphocytic leukemia, immunoglobulin, mass spectrometry, peptide sequencing

## Abstract

A wide variety of immunoglobulins (Ig) is produced by the immune system thanks to different mechanisms (V(D)J recombination, somatic hypermutation, and antigen selection). The profiling of Ig sequences (at both DNA and peptide levels) are of great relevance to developing targeted vaccines or treatments for specific diseases or infections. Thus, genomics and proteomics techniques (such as Next-Generation Sequencing (NGS) and mass spectrometry (MS)) have notably increased the knowledge in Ig sequencing and serum Ig peptide profiling in a high-throughput manner. However, the peptide characterization of membrane-bound Ig (e.g., B-cell receptors, BCR) is still a challenge mainly due to the poor recovery of mentioned Ig.

Herein, we have evaluated three different sample processing methods for peptide sequencing of BCR belonging to chronic lymphocytic leukemia (CLL) B cells identifying up to 426 different peptide sequences (MS/MS data are available via ProteomeXchange with identifier PXD004466). Moreover, as a consequence of the results here obtained, recommended guidelines have been described for BCR-sequencing of B-CLL samples by MS approaches.

For this purpose, an in–house algorithm has been designed and developed to compare the MS/MS results with those obtained by molecular biology in order to integrate both proteomics and genomics results and establish the steps to follow when sequencing membrane-bound Ig by MS/MS.

## INTRODUCTION

The immune system has the capacity to produce a vast repertoire of immunoglobulins, (Ig) in response to the wide number of existing antigens, by processes such as V(D)J recombination (it occurs during B-cell maturation) [1, 2], somatic hypermutation (generated during B-cell affinity maturation) [3, 4] and antigen selection (during B-cell activation) [5].

The Ig structure comprises four chains: two identical heavy chains and two identical light chains linked by disulfide bonds. Heavy chains are classified according to the heavy chain into 5 groups: α, δ, ε, γ, and μ (for IgA, IgD, IgE, IgG, and IgM, respectively). Concerning light chains, there are two types: kappa (κ) and lambda (λ). In turn, each heavy and light chain presents a constant (C) and a variable (V) region. The effector function of the Ig is located in the C region of the heavy chain. The Igs also comprise fragment antigen binding (Fab) and fragment crystallizable (Fc) regions, where Fab region is the part which binds to antigens (including the variable domain of each heavy and light chain) and presents a set of complementary determining regions (CDR), also known as hypervariable regions, located between stable regions named frameworks (FR). In turn, the Fc region is the binding site for endogenous receptors (presented in macrophages, dendritic cells, and other cells of the immune system) and complement system proteins [6].

Igs are present as soluble proteins in proximal body fluids (serum, synovial fluid, and saliva, among others) or membrane-bound proteins attached to the B-cells (similar structure to IgM) for regulating the immune system and performing the function of B-cell receptors (BCR), respectively. Specifically, the disrupted activation of the BCR appears as the responsible of the chronic lymphocytic leukemia (CLL) [7, 8]. This disease, characterized by its high heterogeneity, is the most common human blood cancer in Western countries and it usually shows a monoclonal expansion of an aberrant B-cell clone [9, 10].

Determining the protein profiles of B-CLL cells could have a great impact on disease knowledge, progression, origin, and identification of new drug targets; particularly referring to immune-system proteins, antibodies and immunoglobulins. In this sense, high-throughput DNA sequencing has been the approach of choice for extracting the most Ig biological information [11]. In fact, Next Generation Sequencing (NGS) allows the characterization of millions of BCR sequences in a single experiment [12]. However, despite the remarkable advances made in the field, the peptide sequence information is required for the complete description of the Ig sequences as the cellular state is finally defined by the translated genes (i.e., proteins) – everything that is transcribed (genomics) may not be translated (proteomics)-. Henceforth, the integration of genomics and proteomics data sets for Ig sequencing might provide complementary information about evidence of gene expression at the protein level [13, 14, 15].

The determination of the length and peptide sequence of the H-chain CDR-H3 region (related to antibody specificities) as well as the IGHV and IGHJ gene patterns and the VH:VL pairing [16, 17] are paramount for antibody clonotyping. In fact, the alterations in these sequences could be specific and characteristic of the pathology highlighting the importance of their determination as previously reported by Henriques and colleagues [18]. Moreover, the evaluation of immune repertoires is coming up as a useful approach for the identification of antigen-specific BCR, and evaluation of the efficacy of immune checkpoint blockers and cancer immune therapy, among others [19, 20].

Also, these described approaches are becoming quite critical in the characterization of pharmaceutical IgGs, such as anti-CD20, trastuzumab, and cetuximab [21]. Related to this, the production of biosimilars (biologic medical products which are almost identical copies of original products), which are considered as good alternatives to improve healthcare access and outcomes at reduced costs, could be greatly improved if disease-related antibody sequences are determined (e.g., monoclonal antibodies, cytokines…) [22, 23].

Generally, Ig-sequencing (soluble Ig) studies have been performed by using MS/MS approaches in serum samples [24], whereas BCR-sequencing (membrane-bound Ig) has been addressed from a genomic point of view using B-cells as a sample [25]. Thus, their combination might have a tremendous importance to understand the humoral antibody responses.

Peptide analysis of BCR sequences remains a challenge mainly due to the drawbacks in its processing and purification (e.g., low BCR levels). Thus, NGS approaches have been the usually selected strategies for the amplification of the BCR and its sequencing. However, a library including the mass spectra for BCR-related peptides may be of great interest for the full characterization of BCR production, Ig secretion, and immune responses. Since little variations in antibody sequences (even one single amino acid) could lead to modified specific antigen bindings; then, MS/MS strategies seem a good alternative for providing confidence in peptide sequencing [25].

**Figure 1:**
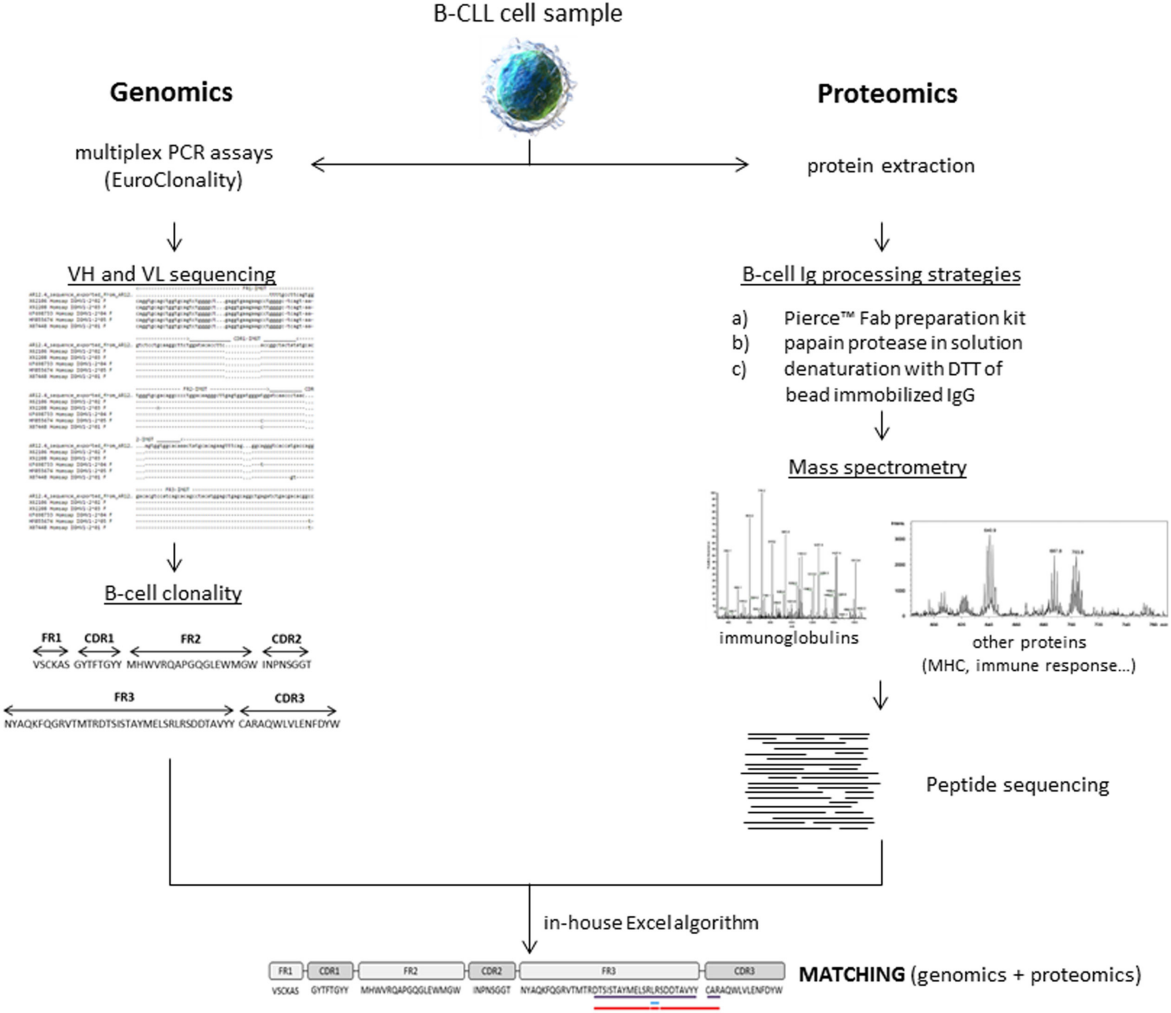
General workflow of the study

To this end, we have evaluated three approaches for peptide sequencing of BCR as well as B-cell proteins associated to BCR (MHC-I, MHC-II, CD20, CD79b, among others) and also related to the immune system using an MS/MS approach. Moreover, an in-house algorithm has been designed for the comparison of sequences obtained by MS/MS and molecular sequencing (Figure 1) to demonstrate the potential of MS/MS approaches in B cells to characterize Ig sequences (426 unique peptide sequences have been identified in this study) and correlate them with DNA sequence libraries.

## RESULTS

### Sample processing for BCR profiling by MS/MS assays

As mentioned in the Introduction section, cellular Igs use to be characterized by DNA-sequencing strategies whereas soluble Igs (due to their high concentration in serum) are easier to be characterized by MS approaches. The key point is the isolation and enrichment of BCR (membrane-bound Ig) in order to increase the peptide coverage that facilitates the peptide sequencing of the BCR. With this purpose, three different approaches (termed Fab preparation kit (a), papain (b), and DTT (c) in Materials and Methods section) were tested to evaluate the utility and robustness of MS/MS assays for Ig peptide identification.

While the first approach (Fab purification kit) uses an available commercial kit for the isolation of Fab fragments from protein samples employing affinity columns, the second and third approaches (papain and DTT, respectively) include an Ig-enrichment step – based on protein G microbeads - previous to the addition of papain or DTT for the isolation of Fab fragments or the breakage of disulfide bonds, respectively. With all these strategies, it is expected to purify the B-cell Igs in order to increase the peptide coverage and the subsequent BCR-peptide characterization.

In Supplementary Information 1, the proteins and the corresponding peptides for each approach are collected. As it has been depicted in Table 1, CLL samples have been randomly distributed among the 3 suggested approaches. Additionally, human IgG protein (whole molecule) was processed using the Fab preparation kit to use it as a reference.

**Table 1:** Patient characteristics

Sample ID	Age^a^ (yr)	Gender	Diagnosis^b^ % clonal population (from WBC)^b^ V(D)J rearrangement^c^	MS^c^ Rai stage	Light chain^b^	Molecular biology sequence	MS/MS processing approach^d^ Gel section digested
1	83	F	CLL 88.1VH4-34(D5-12)J4	UM-HIII	kappa^+dim^	Avail.	DTTWhole lane
2	58	F	CLL 21.4VH3-7(D2-15)	M-H0	kappa^+dim^	N.A.	DTTWhole lane
3	62	F	CLL 22.1VH4-39(D2-2)	UM-HI	kappa^+dim^	Avail.	DTTWhole lane
4	48	M	CLL 90.0VH3-30(D2-15)J6	M-HI	kappa^+dim^	Avail.	DTTWhole lane
5	72	F	CLL 70.1VH3-7(D2-2)	M-HIII	kappa^+dim^	N.A.	DTTWhole lane
6	49	M	CLL 80.4VH1-69(D3-10)J6	M-HIV	kappa^+dim^	N.A.	Fab preparation kitGel band
7	65	M	CLL 87.5VH1-69(D2-15)J3	UM-HN.A.	kappa^+dim^	N.A.	PapainWhole lane
8	70	M	CLL 64.9VH5-a(D5-12)	M-HII-H	kappa^+dim^	N.A.	PapainGel band
9	56	M	CLL 83.2VH1-69(D2-2)J6	UM-HII	lambda^+dim^	N.A.	DTTWhole lane

Clinical characteristics of the samples used in this study, including the age, gender, diagnosis, V(D)J rearrangement, molecular biology features, and MS/MS processing approach applied to each sample.

^a^ Age at time of sample collection. ^b^ From phenotypic studies. ^c^ From molecular biology studies. ^d^ *DTT* for denaturation of bead-immobilized immunoglobulins (Ig); *papain* for isolation of Fab fragments from bead-immobilized Ig; *Fab preparation kit* for isolation of Fab fragments using a commercial kit.

WBC, white blood cells. MS, mutational status (UM-H, unmutated; M-H, mutated). F, female. M, male. CLL, chronic lymphocytic leukemia. Avail., available. N.A., information not available.

A total of 31 different peptides related to Ig were identified when processing the human IgG sample, corresponding to 20 different proteins. As expected, the approach concerning Fab preparation kit allowed the isolation of these peptides from a purified Ig sample. However, its application on a complex sample (purified B-CLL cells, sample 6) reported 22 different proteins (related to BCR and Ig) in comparison with the control sample. Nevertheless, when looking for the 20 human IgG proteins mentioned above in the samples processed with papain and DTT, the overlapping was increased up to 40% (8/20) and 70% (14/20), respectively.

Considering as reference these results, the denaturation approach provides better results when dealing with high complex samples. Nonetheless, and due to this complexity, the purification and enrichment of the sample may become a key step during the processing.

Our results show that the number of identified proteins related to the immune system is increased as the processing strategy is simplified. Thus, 40, 54, and 171 immune system proteins were reported in CLL samples for Fab preparation, papain, and DTT approaches, respectively (Supplementary Information 1). Therefore, for MS/MS characterization of cellular Ig, two main considerations are needed: i) enrichment step for the isolation and purification of Ig-related proteins, and ii) simple post-processing for minimizing loss of sample.

**Figure 2:**
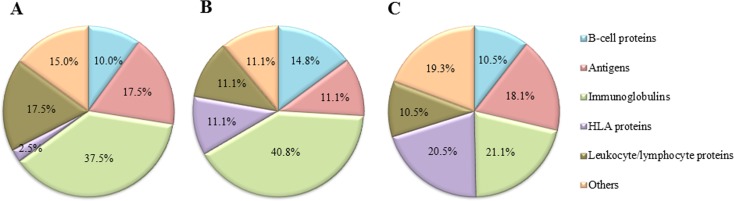
Distribution of proteins related to the immune system detected by shotgun MS/MS after processing the samples by three different approaches **(A)** *Fab preparation kit* for isolation of Fab fragments using a commercial kit; **(B)** *papain* for isolation of Fab fragments from bead-immobilized immunoglobulins (Ig); **(C)** *DTT* for denaturation of bead-immobilized Ig). The MS/MS analysis was performed using Peptide Shaker search engine (neXtProt database, 2016).

Regarding the distribution of immune system related proteins reported by the three approaches (Figure 2), papain approach was the most efficient for the identification of specific Ig proteins (40.8%), followed by Fab preparation and DTT approaches. Furthermore, proteins belonging to the human leukocyte antigen (HLA) system were better detected by the DTT approach (20.5%) in comparison to papain and Fab preparation strategies (11.1 and 2.5%, respectively). The remaining protein groups (B-cell proteins, antigens, leukocyte/lymphocyte proteins, and others) presented a similar distribution.

### Characterization of Ig peptide sequences by MS/MS

The construction of peptide sequence libraries for soluble Igs and BCRs is still a challenge. As mentioned before, several improvements have been done in serum-Ig sequencing, but profiling cellular Ig without using DNA-approaches is really difficult mainly due to the low amount of membrane-bound Ig present in the cell (compared to the rest of cell proteins).

In our study, a total of 60, 98, and 426 different unique peptides were identified across the three approaches (Fab preparation, papain, and DTT, respectively) establishing the beginning of a CLL peptide sequence library exclusively obtained by MS/MS technique. Only 15 peptides out of the total identified were in common among the studied strategies, whereas the approach concerning denaturation of Ig enriched samples reported 362 unique peptides not identified by the other two techniques (data not shown). These results suggest the importance of perfectly well-defined workflow when processing complex samples for Ig sequencing.

**Figure 3:**
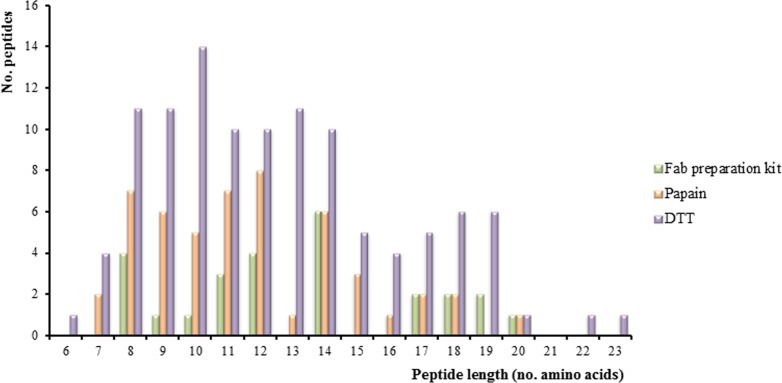
Peptide length distribution Graphic showing the number of peptides corresponding to immunoglobulin (Ig) proteins detected by the three approaches (Fab preparation kit for the isolation of Fab fragments using a commercial kit, papain for isolation of Fab fragments from bead-immobilized Ig, and DTT for denaturation of bead-immobilized Ig) using Peptide Shaker as search engine and neXtProt (release February 2016) as database.

As Figure 3 shows, the peptide sequence length is distributed from 6 to 23 amino acids in the samples. This distribution is more uniform for peptides obtained with the DTT approach. However, the Fab preparation kit has generated a low number of long unique peptides which could be a drawback for the identification of cellular Ig.

Regarding peptide sequence variability, the combination of different proteases (in addition to trypsin, which has been used in this study) might be an important improvement. In this way, peptide sequence libraries could increase in size providing a better Ig characterization.

### Matching genomic and proteomic data sets for BCR profiling at peptide level

Sequencing of membrane-bound Ig of clonal B-cell populations belonging to CLL patients is routinely done. This sequencing is performed by using molecular biology techniques and allows the stratification of the patients, being also crucial for determining the patient treatment. Despite multiplex PCR assays are performed to determine the B-cell clonality (following the guidelines of the EuroClonality European Consortium, http://www.euroclonality.org/); this approach presents a limitation because it is not possible to characterize the whole set of proteins related to the immune system in a single assay. In this regard, proteomics is able to achieve the Ig characterization at peptide level with the same processing speed and robustness as genomics, but with the gain of sequencing thousands of peptides at once (including Ig or proteins related to the immune response, among others). This is of great relevance given the importance of surrounding proteins that determine the final behavior of B-CLL cells.

In fact, both disciplines must be considered as complementary instead of competitive or encountered methodologies. With the purpose of integrating the results from genomics and proteomics, an in-house algorithm in Excel software (Supplementary Information 2) was designed. This tool allows the identification of sequences overlapping between the peptide and DNA-translated providing information about which specific Ig region (CDR or FR) presents the overlapping and the coverage percentage for each sample.

In this study, information about the Ig sequences from three samples (samples ID 1, 3, and 4) are reported and compared to our MS/MS peptide sequences (from the same samples) using the mentioned algorithm.

As Ig sequences are characterized by their high variability, the common databases used for MS/MS searching do not include all of them (hence specific databases only focused on Igs are dramatically needed). Thus, two different search engines (i.e., Mascot and PeptideShaker) were selected for evaluating their capability on detecting non-assigned peptides (i.e., amino acid sequences belonging to highly variable proteins). On one side, PeptideShaker only presents as result sequences matching with annotated unique peptides and their corresponding proteins; whereas the Mascot search engine reports well-defined peptides although they do not correspond to annotated proteins. This comparison will highlight the relevance of the appropriate search engine (independently of the sample preparation) according to the sample features.

The peptides identified by both search engines are collected in Supplementary Information 3 for the three samples mentioned above (“List of peptides” sheet). For Mascot results, “annotated” and “unknown” proteins are displayed in different columns. These peptide sequences were compared to the corresponding molecular biology sequence by using the in-house Excel algorithm. The results of these comparisons are also depicted in Supplementary Information 3.

**Figure 4:**
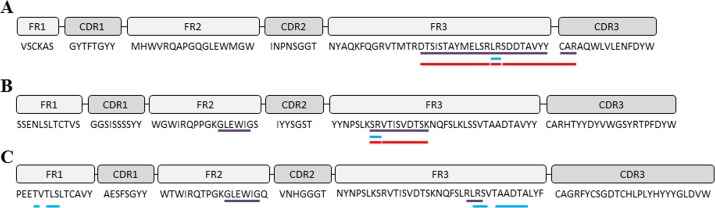
IGH gene sequences The framework (FR) and complementary determining regions (CDR) of IGH genes were analyzed and translated into amino acids after DNA-sequencing using the IMGT tool. **(A)** sample ID 1; **(B)** sample ID 3; **(C)** sample ID 4. Violet lines depict the sequence coverage by both DNA-sequencing and MS/MS sequencing using Mascot search engine (UniProt database). Blue lines correspond to sequence coverage by both DNA-sequencing and MS/MS sequencing using Peptide Shaker search engine (neXtProt database). Red lines correspond to predicted tryptic peptides determined by PeptideCutter tool (ExPASy Bioinformatics Resource Portal).

Figure 4 summarizes the overlapping between both genomics and proteomics and both Mascot and PeptideShaker. As indicated in this Figure 4, FR regions present the greater overlap between genomics and proteomics. Regarding the CDR3 region, 3 amino acids (CAR) have been properly detected (high ion score) within an 11-amino acids length peptide constituting a 20% coverage of the mentioned region.

Regarding the search engine employed, Mascot seems to be more efficient than PeptideShaker. In Table 2, it is reported the coverage percentage for each sample and Ig region. Although PeptideShaker coverage for sample 4 is good enough, Mascot is more homogeneous along the three samples, possibly due to the fact previously mentioned.

**Table 2:** Sequence coverage of IGH genes after DNA-sequencing and MS/MS sequencing and analysis by Mascot and Peptide Shaker search engines

Sample ID^a^	Search engine^b^	Region^c^	Region coverage^d^ (%)	Total coverage^e^ (%)
**1**	Mascot	FR3	23/37 (62.1%)	26/91 (28.6%)
		CDR3	3/15 (20.0%)	
	Peptide Shaker	FR3	2/37 (5.4%)	2/91 (2.2%)
**3**	Mascot	FR2	6/17 (35.3%)	17/104 (16.3%)
		FR3	11/37 (29.7%)	
	Peptide Shaker	FR3	2/37 (5.4%)	2/104 (1.9%)
**4**	Mascot	FR2	6/17 (35.3%)	9/109 (8.3%)
		FR3	3/37 (8.1%)	
	Peptide Shaker	FR1	4/14 (28.6%)	13/109 (11.9%)
		FR3	9/37 (24.3%)	

^a^ Sample ID information is available at Table 1. ^b^ Mascot search engine was used with UniProt database, whereas Peptide Shaker was tested with the neXtProt database. ^c^ It is referred to the framework (FR) or complementary determining region (CDR) of the IGH gene sequence. ^d^ Region coverage was calculated considering the number of amino acids in common between DNA-sequencing and MS/MS sequencing in relation to the region (FR or CDR) length. ^e^ Total coverage was calculated considering the total number of amino acids in common between DNA-sequencing and MS/MS sequencing of the whole IGH gene in relation to the IGH gene length.

## DISCUSSION

The high diversity present in gene sequences requires tailor-made DNA sequence databases, particularly in encoding genes for Ig and histocompatibility complexes. Next-generation sequencing approaches (genomics) have allowed a significant improvement in this area (BCR- and Ig-sequencing), but the progress in proteomics for the BCR-sequencing is still in their beginnings. Whereas the sequencing of soluble Ig is perfectly addressed by MS/MS strategies, the peptide profiling of membrane-bound Ig is still a great challenge.

In this study, we have characterized Ig attached to the B-CLL cells surface at peptide level by performing three different sample preparation approaches (i.e., usage of a Fab preparation commercial kit, and usage of papain in solution and DTT in Ig-enriched agarose beads from CLL protein samples for the isolation of Fab and denaturation of the Ig, respectively). Our results have indicated that the strategy including denaturation with DTT is the most efficient approach. Bearing in mind these results, several guidelines could be described about peptide sequencing of membrane-bound Ig: i) including an Ig-enrichment step; ii) reducing the complexity of the procedure (with regard to the number of steps) to avoid the loss of the sample (membrane-bound Ig are less abundant than soluble ones); iii) using a suitable MS/MS search engine for the identification of peptides corresponding to uncharacterized or unassigned proteins; and iv) integrating MS/MS and molecular biology results to get a wide and comprehensive view of Ig and associated immune system proteins expressed in response to a specific disease or particular cellular condition.

In summary, all these results indicate that the integration of genomics and proteomics could provide a full characterization of Ig sequences, BCR and the proteins related to the immune system in response to a specific disease or particular cell situation. Nonetheless, further assays will be required to identify specific patterns in BCR sequences which could be of interest in pathological situations such as CLL, autoimmunity or immunodeficiency, among others.

## MATERIALS AND METHODS

**Figure 5:**
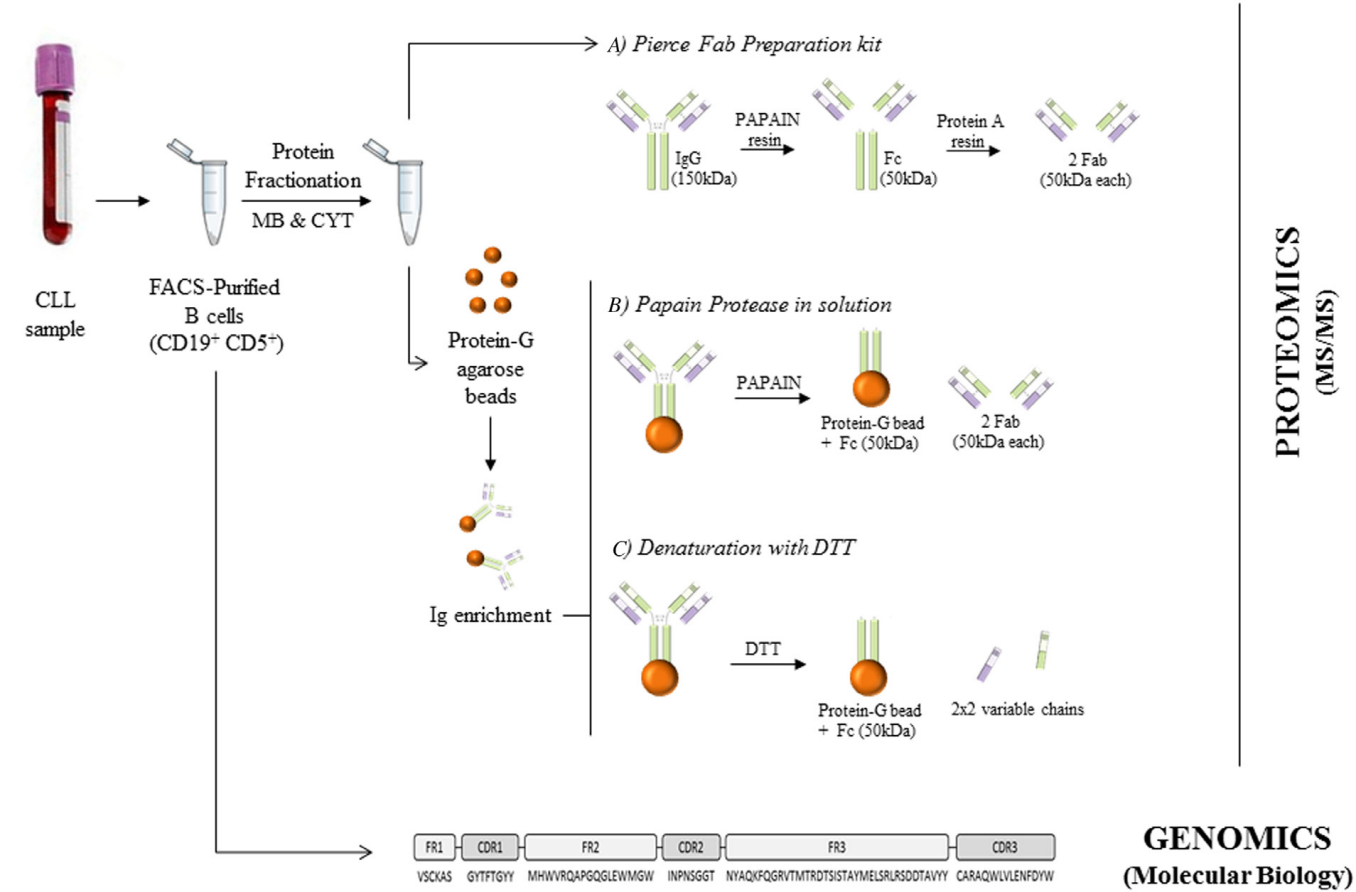
Overview of the procedures performed in this study B cells were purified from CLL peripheral blood using FACSAria. These purified cells were directly employed for BCR-sequencing by molecular biology and also processed to obtain membrane (MB) and cytoplasmic (CYT) proteins. These isolated proteins were used for Fab purification using Pierce Fab preparation kit **(A)**. Moreover, they were Ig-enriched for applying papain protease in solution **(B)** and denaturation with DTT **(C)** for the BCR-sequencing by LC-MS/MS.

Figure 5 depicts a general overview of the procedure described below.

### Sample collection

Peripheral blood (PB) B-lymphocytes (CD19^+^ CD5^+^) from 9 CLL patients (Table 1) were systematically purified by FACSAria II flow cytometer (BD, San Jose/CA, USA) (at a purity >95%) at General Service of Cytometry of University of Salamanca [26]. High-sensitive multi-parameter flow cytometry was performed for the immunophenotyping of clonal B-cell populations, according to previously described procedures [18]. All cases presented a clonal (8 kappa CLL clone, 1 lambda CLL clone) and aberrant CD5^+^ CLL-like B-cell population.

PB samples were obtained with informed consent and according to the guidelines of the local ethics committees of the University Hospital of Salamanca, in accordance with the Helsinki Declaration of 1975, as revised in 2008.

### Molecular studies: sequencing of immunoglobulin human domains

Each clonal B-cell population – purified by FACSAria – was screened for its patterns of rearrangement of the IGHV genes (Table 1) following genomic DNA preparation, PCR amplification, sequencing, and analysis of V, (D), J genes, as described elsewhere [27, 28]. Specifically, the B-cell clonality was determined following the guidelines described by the EuroClonality Consortium, http://www.euroclonality.org/, which combine the usage of IGH and IGK multiplex PCR assays for the assessment of Ig gene rearrangements [29, 30]. The sequences of the V(D)J fragments of these samples have been widely reported in a previous study performed by our group [31]. Resulting sequences were aligned with germline sequences using the IMGT database and tools (http://www.imgt.org). Sequences were considered as somatically mutated if they contained 2% deviation from the germline sequence.

### Subcellular protein fractionation

FACS-purified B cells were snap-frozen in liquid nitrogen after adding lysis solution (5 mM HEPES, 10 mM MgCl_2_, 140 mM NaCl, 0.1% Tween 20, and 1% protease inhibitor mix. All purchased from Sigma, St. Louis/MO, USA). Then, cells were thawed on ice and centrifuged at 15,000 g for 15 min; henceforth, supernatant and pellet were treated separately, although processing stepwise in an identical manner. 10 % (w/v) octyl-β-D-glucopyranoside (Sigma) was added followed by sonication (3 pulses for 3 seconds each). Samples were incubated for 30 min and centrifuged at 15,000 g for 15 min. Cytoplasmic proteins were collected from the supernatant, whereas membrane proteins were collected from the pellet.

### Immunoglobulin enrichment from cell extract

Samples were IgG-enriched by using protein-G-agarose beads (GE Healthcare, Buckinghamshire, UK), according to manufacturer's instructions, and stored at -80°C until used.

### B-Cells immunoglobulin processing strategies

Three different approaches were performed to achieve the peptide sequencing of B-cell Igs: a) Pierce™ Fab preparation kit (Thermo Scientific, Waltham/MA, USA), based on the usage of immobilized papain protease for IgG digestion and protein A agarose for purification; b) papain protease in solution (Worthington, Biochemical Corporation, Lakewood/NJ, USA), for the digestion of bead immobilized IgG and the obtaining of Fab fragments [32]; c) denaturation with DTT of bead immobilized IgG for the breakage of their disulfide bonds and the obtaining of heavy and light chains.

Approach a) was performed following the manufacturer's indications. For approach b), papain was activated with a digestion buffer (5 mM L-Cys-HCl, 1 mM EDTA and PBS; all from Sigma) at pH=7 for 30 min at 37°C. The activated papain was incubated with the sample at 1:50 ratio (papain:sample) for 2 h at 37°C with gently mixing. Afterward, samples were centrifuged at 12,000 g for 3 min and the supernatant was collected. The supernatant contained the Fab released from the IgG, whereas the pellet contained the beads and Fc remaining fractions. Papain digestion was stopped with SDS sample buffer. The second step of papain digestion was performed with the pellet at the same conditions indicated above and joined to the one obtained in the first digestion. Finally, approach c) was performed incubating the samples with 40 mM DTT for 5 min at 99°C.

In all these performed approaches (a, b, and c), human IgG protein (Abcam, Cambridge/UK) was used as a reference.

### SDS-PAGE separation

Each processed sample was separated on a 10% SDS-PAGE gel under non-reducing conditions for approaches a) and b), and reducing conditions for approach c). After electrophoresis, gels were stained in a solution of 2.5 g/L silver nitrate and stored at 4°C in an aqueous solution containing 1% (v/v) acetic acid, until analysis (Supplementary Figure 1).

### nUPLC-MS/MS analysis

Each gel lane was cut into fragments and digested with trypsin following the method of Shevchenko *et al*. [33] with slight modifications. Briefly, gel pieces were destained with 15 mM potassium ferrocyanide and 50 mM sodium thiosulfate. Protein reduction and alkylation were performed with 10 mM DTT at 56°C for 45 min, and with 55 mM IAA at room temperature for 30 min, respectively. Proteins were digested with trypsin (6.25 ng/mL) at 37°C for 18 h. The peptide solution was acidified with FA and desalted by using C18-Stage-Tips columns [34]. The samples were partially dried and stored at −20°C until they were analyzed by LC-MS/MS.

A nUPLC system (nanoAcquity, Waters Corp., Milford/MA, USA) coupled to a LTQ-Velos-Orbitrap mass spectrometer (Thermo Fisher Scientific, San Jose/CA, USA) via a nanoelectrospray ion source (NanoSpray flex, Proxeon, Thermo) was used for reversed-phase LC-MS/MS analysis. Peptides were dissolved in 0.5% FA/3% ACN and loaded onto a trapping column (nanoACQUITY UPLC 2G-V/M Trap Symmetry 5 μm particle size, 180 μm × 20 mm C18 column, Waters Corp., Milford/MA, USA). Peptides were separated on a nanoACQUITY UPLC BEH 1.7 μm, 130 Å, 75 μm × 250 mm C18 column (Waters Corp., Milford/MA, USA) with a linear gradient from 7% to 35% solvent B (ACN/0.1% FA) at a flow rate of 250 nL/min over 120 min.

The nUPLC- LTQ-Orbitrap Velos was operated in the positive ion mode by applying a data-dependent automatic switch between survey MS scan and tandem mass spectra (MS/MS) acquisition. Survey scans were acquired in the mass range of *m*/*z* 400 to 1600 with a 60 000 resolution at *m*/*z* 400 with lock mass option enabled for the 445.120025 ion [35].

The 20 most intense peaks having ≥2 charge state and above the 500 intensity threshold were selected in the ion trap for fragmentation by collision-induced dissociation with 35% normalized energy, 10 ms activation time, *q* = 0.25, ± 2 *m*/*z* precursor isolation width and wideband activation. Maximum injection time was 1000 and 50 ms for survey and MS/MS scans, respectively. AGC was 1 × 10^6^ for MS and 5 × 10^3^ for MS/MS scans. Dynamic exclusion was enabled for 90 s.

### Database search

Raw data were translated to mascot general file (.mgf) format using Thermo Scientific Proteome Discoverer software (v. 1.4.1.14). The searches were conducted using both Mascot and Peptide Shaker search engines. For the first approach, the MASCOT [36] algorithm was used to search for the acquired MS/MS spectra, using Thermo Scientific Proteome Discoverer software (v. 1.4.1.14) against a custom database of all human-reviewed sequences downloaded from the UniProt database (February 2014) and common contaminant sequences (e.g., human keratins, trypsin, BSA). Search parameters were as follows: fully-tryptic digestion with up to two missed cleavages; 10 ppm, and 0.8 Da mass tolerances for precursor and product ions, respectively; carbamidomethylation of cysteines as fixed modification and oxidation of methionine and n-terminus acetylation as variable modifications were considered. Peptides having MASCOT ion scores of <20 were not considered for analysis. A 1% false discovery rate (FDR) using Percolator [37] was employed for peptide validation. For Peptide Shaker approach, the search was conducted using SearchGUI version 1.30.1 [38]. Protein identification was conducted against a concatenated target/decoy version of all human-reviewed sequences downloaded from the neXtProt database (release of September 2015, 20066 protein entries) and common contaminant sequences (e.g., human keratins, trypsin). The decoy sequences were created by reversing the target sequences in SearchGUI. The identification settings were the same fixed for the MASCOT searching. Peptides and proteins were inferred from the spectrum identification results using PeptideShaker version 0.41.1 [39]. Peptide-Spectrum Matches (PSMs), peptides, and proteins were validated at a 1% FDR estimated using the decoy hit distribution.

The MS data along with the identification results have been deposited to the ProteomeXchange Consortium via the PRIDE [40] partner repository with the dataset identifier PXD004466.

### Peptide mass spectra and DNA sequences interpretation

An in-house Excel algorithm was designed for the integration of results obtained after DNA sequencing and MS/MS analysis. The basis of this Excel macro was the searching of homologies between the translated DNA sequences and the peptides identified by nUPLC-MS/MS. This tool looks for homology of 2 or more consecutive amino acids corresponding to the CDR or FR regions. For reviewing purposes, the Excel algorithm is provided as Supplementary Information 2.

## SUPPLEMENTARY MATERIALS AND FIGURE









## Abbreviations

BCR: B-cell receptor
CDR: complementary determining region
CLL: chronic lymphocytic leukemia
Fab: fragment antigen binding
FR: framework
HLA: human leukocyte antigen
Ig: immunoglobulin
MS: mass spectrometry
NGS: Next-Generation Sequencing
nUPLC-MS/MS: nano-ultra-performance liquid chromatography tandem MS
PB: peripheral blood
PSM: peptide-spectrum match
