# Supplementary material for: A systematic approach for peptide characterization of B-cell receptor in chronic lymphocytic leukemia cells
Source: Oncotarget. 2017 Apr 13;8(26):42836–46. doi: 10.18632/oncotarget.17076 (PMC5522109; doi:10.18632/oncotarget.17076)
Supplement: Supplementary file 1 [file oncotarget-08-42836-s001.pdf]

A systematic approach for peptide characterization of B-cell receptor in chronic lymphocytic leukemia cells

SUPPLEMENTARY INFORMATION

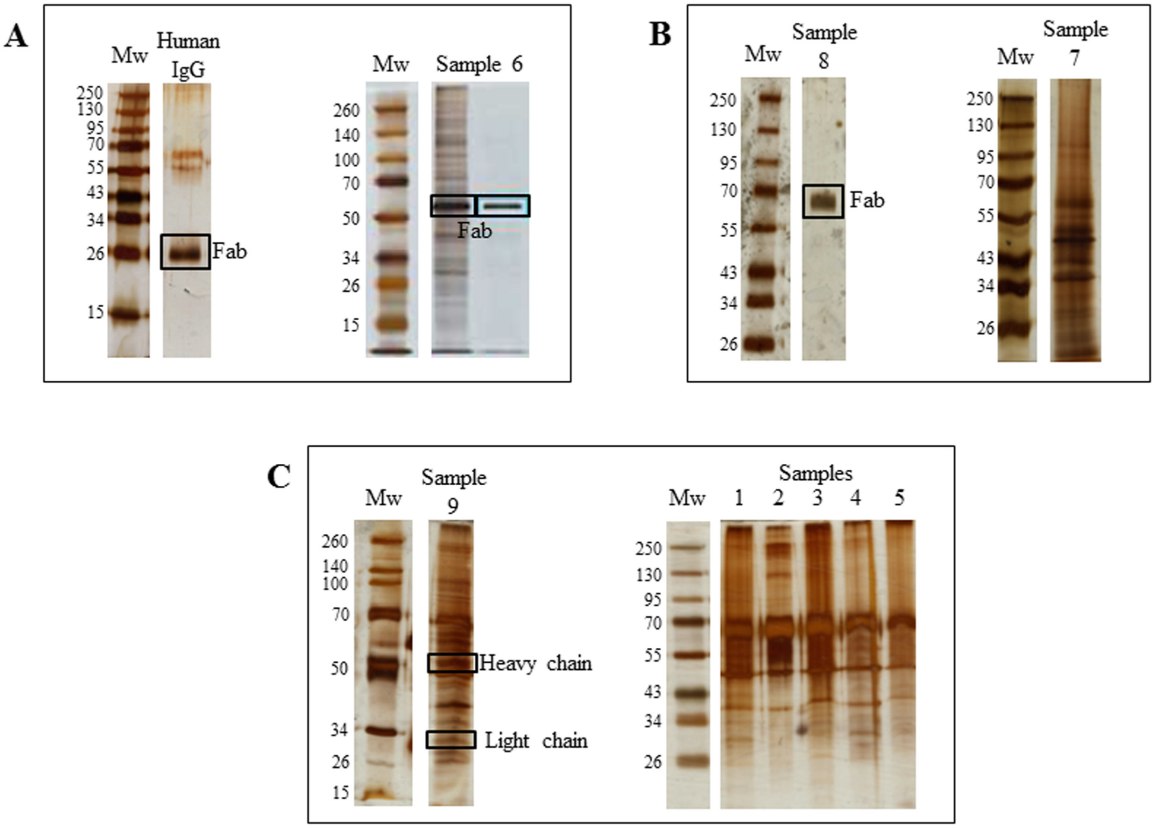

Supplementary Figure 1: Silver-stained SDS-PAGE gels corresponding to the samples processed following Fab preparation kit approach **(A),** papain approach **(B),** or DTT approach **(C)**.

Supplementary Information 1: List of proteins and peptides related to the immune system identified throughout the three processing approaches Fab preparation kit (1st sheet), papain approach (2nd sheet), and DTT approach (3rd sheet)

See Supplementary File 1

Supplementary Information 2: In-house Excel algorithm designed for the *in silico* comparison of IGH sequences obtained by using both DNA-sequencing and MS/MS sequencing (after denaturation of bead-immobilized immunoglobulins)

The file contains two sheets: *Data*, for the IGH sequences from molecular biology and MS/MS strategies; *Results*, where the sequence overlapping is shown.

See Supplementary File 2

Supplementary Information 3: List of peptides corresponding to Ig and immune system proteins identified for samples 1, 3, and 4, and comparison with the IGH gene obtained by molecular biology

"List of peptides" sheet for each sample and each search engine (Mascot or PeptideShaker), differentiating between known and unknown proteins from Mascot. "Predicted tryptic peptides" includes the list of possible peptides obtained after *in-silico* trypsin digestion with ExPASy PeptideCutter tool for each sample (IDs 1, 3, and 4). The remaining sheets include the results obtained after using the in-house Excel algorithm (Supplementary Information 1) with the list of peptides present in "List of peptides" sheet. In red are shown the amino acids/peptides detected in common by both molecular biology and MS/MS shotgun.

See Supplementary File 3
